# Hospital Construction in Germany
**Modern German Hospital Construction.* By William Milburn, jun., A.R.I.B.A. Royal Institute of British Architecta, 9 Conduit Street, Regent Street, London, W. 1912. Price 3s. 6d. net.


**Published:** 1912-08-17

**Authors:** 


### HOSPITAL ARCHITECTURE AND CONSTRUCTION.

[Communications on this subject should be marked "Architecture" in the left-hand top corner of the envelope.]

### Hospital Construction in Germany.*

SOME CRITICISMS AND AN APPRECIATION BY A BRITISH ARCHITECT.

Mr. Milburn has been well advised to issue in pamphlet form a reprint of his excellent series of articles contributed to the *Journal of the Royal Institute of British Architects* (3rd Series, Vol. xix., Nos. 2, 3, and 4), in which he discussed modern German hospitals from the architect's point of view. His qualifications as a critic are indisputable. Mr. Milburn was Henry Saxon Snell prizeman in 1908 and Godwin Bursar in 1910, and his tour in Germany was made in the former capacity for the express purpose of familiarising himself with the type of institution that is favoured by German hospital constructors. He has had the assistance of the foremost German architects, whose help he fully acknowledges. As a result his articles are up to date, clear, instructive, and informative.

### The Value of the Administrative Expert.

If, before discussing his conclusions, we may hazard a criticism it is that his work would have gained in value had he at the same time familiarised himself with the views of such hospital experts as Herr Diesener, Herr Einert, who has charge of the fine Dresden hospitals, Geheimrat Dr. Credé, and Hofrat Dr. Brunner, who is director of the third municipal hospital at Munich-Schwabing. The opinion of such administrative experts is of great importance to the architect who wishes to work hand in hand with those who are to use the hospitals that he plans. The constructor, in Germany as in England, is only too apt to forget or overlook points which seem to him of minor importance but which to the hospital superintendent are of supreme importance. That is a fact we have always insisted upon, and which Mr. Milburn tacitly admits, for the hospitals that have most favourably impressed him are just those institutions which have been designed by architects who have worked in cordial co-operation with administrative experts.

Mr. Milburn starts his series by a brief and very lucid introductory article which deals with the German system generally and with the principles of hospitalisation as understood in Germany. He points out with what care hospital sites are selected, and shows how the tendency has been to increase the area per bed—a tendency which at once strikes the investigator who compares German with English hospitals. Then he traces the progress of modern hospital construction, starting from the Moabit, which sprang out of the old cholera barracks on the Tempelhofer Fields, with the Eppendorf, built in the late eighties of the last century, and ending with the fine new constructions, such as the Virchow, the Nuremberg General, the Charlottenburg West End, the new Munich, the St. George, Hamburg, and the Düsseldorf hospitals. Everywhere he takes care to give clear plans and helpful illustrations which considerably enhance the value of the articles, and we must congratuate him warmly on the choice of his blocks.

### The Effects of the Unit System.

One notices some omissions, but these are, perhaps, only excusable, and perhaps the most serious of them is to overlook the influence of the unit system. In Germany especially the importance of separate stations—as units are there called—is now well recognised, and though Mr. Milburn illustrates these stations and pays particular attention to certain essential departments, such as the children's ward, the maternity block, and the hydro-therapeutic department, he does not, in our opinion, lay sufficient stress on the modifications which the adoption of this unitary system has brought about in the general scheme of hospital construction. The elaborate use of subways and underground passages, which is a feature in modern German hospitals, he refers to in the paragraphs dealing with the mortuary block, but he scarcely devotes adequate space to a description of such blocks. He figures the Virchow pathological block and gives a photograph of the mortuary chapel attached to that institution—a chapel which, as we pointed out in our description of the hospital some years ago, is needlessly elaborate. He does not figure the fine mortuary block of the new Charité or of the Pankow Hospital, but fully recognises the importance of the general arrangement in these new mortuaries, which fulfil all the requirements that can be demanded by the public and by the medical staff. One point, we think, he might with advantage have included—the provision of bathing and lavatory accommodation in the mortuary block which is characteristic of the newest hospitals.

The administrative block is discussed in some detail. Mr. Milburn animadverts on the undesirability of having a central entrance to these blocks, through which patients are admitted. This is an objectionable feature both from an architectural and from an administrative point of view, but we are afraid that its adoption is largely due to the architects who desire to give to the administrative block an effective frontage. That, too, is perhaps the reason why the administrative blocks of late years have become more and more majestic. The new buildings of the Moabit, for example, are palatial and grand, but it is problematical if the added expense is justifiable when their plans add to the administrative difficulties which the superintendents have to cope with. At Charlottenburg, as Mr. Milburn points out, the common entrance has been replaced by putting the receiving block in a separate wing, which is balanced at the other extremity of the administrative block by the dispensing station.

* *Modern German Hospital Construction.* By William Milburn, jun., A.R.I.B.A. Royal Institute of British Architects, 9 Conduit Street, Regent Street, London, W. 1912. Price 3s. 6d. net.

We are glad to notice that Mr. Milburn has paid particular attention to the administrative blocks of the Munich and Cologne institutions, which in many ways are superior to any others in Germany.

### The Small Ward Tendency.

Coming to the ward designs, Mr. Milburn shows how the tendency has been towards smaller wards, the ideal being a ten-bedded ward which "admits of a thorough classification of the diseases and patients, rendering them much easier to nurse and control, while at the same time the individuality of the single patients can be much better looked after." He deals fully with the infectious diseases pavilions, but hardly devotes enough attention to the interesting private wards, which are usually complete units and are in many cases almost ideal from an administrative point of view. The children's wards he discusses are those at Düsseldorf, which are really excellent and of which he gives a good description of the well-planned isolation room, and the Charité clinic at Berlin, which is almost a model. He also cites the Lindenburg and Frankfort City Hospitals as good models—an opinion with which we cordially agree. The maternity wards that appealed to him are those at Eppendorf, Düsseldorf, Dresden, Hamburg, Frankfort, and Cologne. Our own opinion is that the Göttingen and Greifswald clinics present some points of further interest that will repay study so far as the maternity unit is concerned. "The Munich Royal Psychiatrical Clinic," he states, "is said to be the finest in the world." That is a matter of opinion, but it certainly is not to-day the last word in the construction of mental departments in Germany. Since Kraepelin and Littmann planned it in the early years of the present century much has been done to modify the planning of mental wards, as can be seen in the construction of the newer psychiatrical clinics in Germany. With the Halle institution, however, it is still a most interesting design.

Mr. Milburn notices that one of the prevailing features in German plans is the non-disconnection of the "sanitary rooms by a cut-off lobby." "This," he remarks, "is a point in which the German plans differ greatly from the English, and at first sight appears to condemn the design, but where the rooms are well placed and modern and up-to-date apparatus (is) employed it is difficult to note any great objection to the non-disconnection." With regard to general principles of construction, we may quote some excerpts which are interesting. "Pitched roofs are steel or timber framed and covered with tiles, glazed tiles, as at Munich III. and Dresden, being a favourite material. With regard to the flooring, terrazzo is laid in sections, with brass strips between, so as to prevent cracking. Linoleum is now very largely employed and is greatly favoured. Sometimes, when on an upper floor, it is laid in a bed of cork, so as to deaden the sound. . . . Linoleum is also very largely employed as an inlay to corridors. Wood floors are exceptional, though oakwood block floors are occasionally found. . . . Furnishings are as simple as possible, and often most artistic in design." He deals fully with the theatre unit, and describes the excellent operating rooms at the St. George. We note that he thinks the former "tendency to keep the operation house entirely separate and detached from the pavilions is passing away, and the latest hospitals show it connected to the pavilions by closed corridors, so as to obviate the transport of the patients through the open." That former tendency was the result of careful investigation, since it was shown that the percentage of post-operative broncho-pneumonia was lower in hospitals with disconnected theatres than in those which possessed theatres connected to the surgical block by closed corridors. The modern tendency is entirely towards simplifying the operating theatre as much as possible, and to do away with the elaboration that followed after Mickulitz had built his "modern" theatre at Breslau. For the best types of the modern theatre in Germany the hospital worker should go to some of the smaller and newer institutions, and not to the fine municipal clinics, where everything has been done almost regardless of cost.

### A Department Neglected in England.

Properly Mr. Milburn devotes some attention to the hydro-therapeutic department—a unit which is entirely neglected by English hospitals, which are content to "adapt" their basements for this station, as at Charing Cross. The finest type of this unit in Germany is not found attached to a hospital, but is located in a separate building—the new Hydro-Therapeutic Institute of the University of Berlin. Mr. Milburn gives some good plans and illustrations of the Virchow hydro-therapeutic unit and of that belonging to the St. George Hospital at Hamburg. He deals *in extenso* with the domestic block and with the technical or economic stations, giving everywhere elucidative plans and photographs. Finally he devotes some attention to the important question of ventilation, and discusses lighting, water supply, sanitary apparatus, and drainage. With regard to cost, he shows how greatly German hospitals vary in cost per bed. The Nuremberg Hospital was erected at a total cost of £215 per bed; the Johannstadt at Dresden at a cost of £357; and the Virchow at a cost of £477. The Moabit originally cost only some £60 per bed, but recent reconstruction and rebuilding have brought the cost to a figure nearly equalling that of the Virchow. We congratulate Mr. Milburn on his work, which is of considerable interest and value to every one who is interested in hospitals and their construction. His articles are not comparative, and we would have liked more criticism on several points, but they are intended, we take it, as a general survey of the best and finest German institutions of to-day. As such they have a permanent value both to the architect and to the hospital administrator, for they supply a want, since we possess no other report so full, so clear, and so informative as this short pamphlet. He supplies a useful bibliography at the end of his articles. We look forward to a more critical and more comprehensive series of articles from his pen; for he has shown that he has the necessary qualification for a hospital critic.

